# Techniques for the Management of Failed Surgery for Fractures of the Neck of Femur

**DOI:** 10.2174/1874325001711011223

**Published:** 2017-10-31

**Authors:** Philip M Stott, Sunny Parikh

**Affiliations:** Brighton and Sussex University Hospitals, Eastern Rd, Brighton, BN25BE, England

**Keywords:** Hip Fracture, Revision surgery, Internal fixation, Plate osteosynthesis, Dislocated hip hemiarthroplasty, Complications of surgery

## Abstract

**Background:**

The majority of modern surgical treatments for managing hip fracture in the elderly are successful and result in a very low rate of revision surgery. Subsequent operations are however occasionally necessary. Optimal management of complications such as infection, dislocation or failed fixation is critical in ensuring that this frail patient group is able to survive their treatment and return to near normal function.

**Methods:**

This paper is a discussion of techniques, tips and tricks from a high volume hip fracture unit

**Conclusion:**

This article is a technique-based guide to approaching the surgical management of failed hip fracture treatment and includes sections on revising both failed fixation and failed arthroplasty.

## 1 INTRODUCTION

An increasing incidence of hip fracture worldwide has brought a concomitant burden of surgical fixation of those with an extracapsular fracture pattern. While failure is rare, due to both surgical capability and a high mortality in the year after surgery before the implant has time to fail, should it occur it needs significant surgical expertise to treat.

## 2 THE DISLOCATING HEMIARTHROPLASTY

Hemiarthroplasty prostheses have a large diameter head and hence a high jump distance. This should render them inherently more stable than a total hip replacement [1] but nonetheless they have a dislocation rate as high as 9% reported in the literature [2]. The reasons for this are complex and tend to relate more to the characteristics of the patients, who by merit of having been managed by hemiarthroplasty are, by definition, physiologically less fit and medically more complex are not fit for total hip replacement. Such characteristics may include a propensity to fall, pre-existing contractures, deformities or neuromuscular imbalances due to stroke or Parkinson’s disease and cognitive impairment. A dysplastic hip presents additional problems and a shallow or protrusio acetabulum should be looked for on pre- as well as post-operative radiographs.

Surgical factors include the choice of approach and its repair, the version of the femoral component, the size of the head and the offset of the construct. This will, in turn, affect the soft tissue tension of the repair and joint. Most such cases are performed by surgical trainees under supervision.

A meticulous history is required, which may require assistance from a relative or carer. Was this dislocation anterior or posterior? Did the joint dislocate when the patient was standing upright and externally rotating their leg, or was the hip flexed and internally rotated? Are there any symptoms or signs of infection? The neurovascular function of the leg should be assessed and documented. Any contractures should be noted. A set of blood tests including C-reactive protein (CRP), Erythrocyte Sedimentation Rate (ESR) and white cell count (WCC) is essential to exclude infection [3]. Plain radiographs are required to confirm the dislocation and exclude fractures or disassociation of modular components. Sometimes, gross surgical problems such as a significant leg length discrepancy (LLD) or mal-position can be seen on a plain radiograph [4]. The initial operative note should be consulted to establish which approach was used and whether any difficulties or abnormalities have been documented. Antero-lateral approaches tend to dislocate anteriorly, and posterior approaches tend to dislocate posteriorly [5-7].

### 2.1 Management Options

The patient and relatives or carers should be informed that this is a significant complication with a high risk of mortality and morbidity. In our unit, the first dislocation of a hemiarthroplasty is managed by a closed reduction. This is usually only beneficial when a cause such as a contracture or a fall have been identified [3]. Traction and abduction braces do not maintain reduction of an unstable hemiarthroplasty [8] and in this patient group risk contributing to venous thromboembolism (VTE), pressure damage, chest infection, muscle wasting and urinary tract infections [9]. If adductor contractures are identified as the cause, they can be relatively easily managed with a percutaneous adductor tenotomy.

If the patient dislocates again and infection has been excluded, three primary options exist. Firstly, the hemiarthroplasty can be left dislocated. This is usually painful for a variable period of time, but the pain will settle to a manageable level. This is more difficult if there is sciatic nerve compromise from the head of the hemiarthroplasty [10], and we advocate it only where fitness for anaesthesia precludes any surgical option.

Excision arthroplasty is a rapid operation when performed by an experienced surgeon and a cemented femoral component may usually be removed whilst leaving the mantle intact [11]. This is our option of choice for a patient fit for a short operative procedure but not for exploration and revision of a failing hemiarthroplasty.

Exploration should, if excision is not planned, be an operation which may proceed to the point where a stable joint is achieved through as many revision steps as are necessary. The strategy should hinge on this operation being their last on this hip [12]. The steps in this surgery depend upon the original surgical approach.

### 2.2 Previous Posterior Approach

The old wound and the fascia lata are reopened. The sciatic nerve should be inspected and protected – this step may be time-consuming and the nerve heavily encased in scar tissue. The abductor mass (gluteus medius and minimus) should be intact [4]. The posterior repair will have failed. The acetabulum should be checked for fracture, loose bodies and retained cement. If there is a fracture to the wall of the acetabulum, a revision-type cementless acetabular component will be needed, whereas a column fracture requires internal fixation or bypassing [13].

Head trials should be used to verify that the correct head size has been used. The hip should be carefully reduced and cycled through its range of motion. The hip will most likely dislocate in flexion greater than 90 degrees, adduction and internal rotation. This should be checked and recorded. Usually, the dislocation will have been caused by incorrect version of the stem, offset, length or a combination of these [3]. Changing only the length of the head is, therefore, unlikely to succeed. All these problems can be addressed with a cement-in-cement revision of the femoral component, using a prosthesis such as the Exeter™ (Stryker, Illinois) 125mm x 44 stem. This component is designed to be used in residual, intact cement mantles, offers more offset than most hemiarthroplasties and is relatively simple to implant in a timely manner [14]. Hemiarthroplasty heads exist to be used with this component (Unitrax™, Stryker Illinois). While one cementless prosthesis can be revised to another, its rotational stability must be ensured. It may be safer to improve this stability by opting for a cemented implant [15]. Once the rotation, length and offset have been optimised, the hip is reduced with an appropriately sized head. If the hemiarthroplasty is still unstable, either a total hip arthroplasty with a dual mobility or constrained liner or an excision arthroplasty are the remaining viable options [16]. Care is taken when closing the hip to perform a sound posterior repair, closing if possible the labrum, capsule and short external rotators.

### 2.3 Previous Anterolateral Approach

The wound and the fascia lata are reopened. The abductor mass should then be inspected for integrity - most surgeons use a braided, absorbable suture to reattach it during an anterolateral approach. The breaking strain of a typical such suture is less than 200N (20 Kg) [17], far less force than is exerted by the abductors at several times the patient’s body weight. As patients are normally permitted to bear weight fully post-operatively, one of the prime goals of hip fracture surgery, it is common to discover the whole abductor mass has failed and become avulsed from the greater trochanter.

As gluteus medius is the main muscular stabiliser of the hip, it is recommended to proceed straight to a cemented captive acetabular component if the posterior third of the abductor mass is deficient [18]. As in the posterior approach, the acetabulum should be checked for fracture, loose bodies or retained cement, with the same strategies employed for any problems encountered [13].

The head sizing should be assessed, again using trials, and the hip reduced. It should then be tested for stability and will be most likely to dislocate when the leg is straight, adducted and externally rotated. The range of motion and stability should be recorded.

The commonest cause of dislocation seen in our practice is excessive anteversion of the stem. While this is, anecdotally, a common finding, the variability of femoral anteversion in hemiarthroplasty has not been extensively studied. A single small-scale study looked at the ability of surgeons to estimate femoral component anteversion, but this did not go on to attempt to establish an association with the risk of dislocation. This is likely to be due to the large number of cases required to study dislocation risk combined with the requirement for CT scanning.

At revision surgery the femoral anteversion can be reduced to neutral or even slightly retroverted using a cemented stem. The hip can then be reduced again and checked for soft tissue tension and stability. The appropriate neck length is chosen. The hip is reduced. The anterior approach defect in the abductors is repaired with trans-osseus sutures. It is usually reinforced with tenodesis of the tensor fascia lata muscle [19].

## 3 FAILED FIXATION OF EXTRACAPSULAR FRACTURES

The patient’s age, health and ambitions should be taken into account when deciding the best treatment for failed fixation [12]. If a 30 year-old patient had sustained a fracture that was treated with a DHS, but the plate had failed owing to poor reduction, then further attempts should be made at conserving the patient’s femoral head [20]. However, most of these fractures occur in osteoporotic bone. These patients are usually elderly and have multiple comorbidities. The surgeon should try to make sure that this time the operation is successful, and will allow the patient to regain their mobility and independence. Many patients are not strong enough to comply with partial weight-bearing. Often the failed metalwork will have cut out from the femoral head, incurring damage to both the femoral head cartilage and the acetabular socket.

Locking proximal femoral plates are designed for complex proximal fractures. They are load bearing devices which do not allow collapse. If weight bearing is not limited, then these have a high chance of failure [21]. They are therefore not suitable for revision of osteoporotic fractures.

### 3.1 Early Failure (<3 Months)

This occurs before union has occurred. The surgeon should understand why the fracture has failed. Was there a technical issue with the operation, such as inadequate reduction or Tip Apex Distance [22]? Has the biology of the fracture been compromised by an open reduction, or use of a cerclage technique? Was the correct implant used, ie should a nail have been used instead of a DHS? Has the possibility of infection been eradicated? If the surgeon can identify a reason why the fracture has failed, and there is not articular damage on either side of the joint, then it is reasonable to reattempt fixation. There is little point redoing an operation which was technically perfect. If it has failed once, it will be likely to fail again in tissue that has had a previous operation. A suggested algorithm is shown in Fig. (**1**)

### 3.2 Revision Fixation Techniques

The old metalwork is carefully removed through a muscle sparing incision, e.g subvastus. If the lag screw of a DHS was in a good position, then this can be reused. Consider the use of a 150 degree DHS plate which helps ensure a more valgus position of the femoral neck, reducing shear forces acting across the fracture site. The old plate is removed. The fracture is reduced anatomically. The lag screw insertion handle is reattached to the screw and then a new plate is attached. Sometimes it is essential to modify the hole in the lateral wall of the femur, using fine osteotomes or the triple reamer. A compression screw can be used to carefully compress the fracture together [23]. This should be removed before wound closure. If the screw position has to be altered, consider using a device with cement augmentation, e.g PFNA™ (DePuy Synthes).

## 4 TOTAL HIP REPLACEMENT FOR EARLY FAILURE OR NON-UNION

The stability of a hip relies on properly functioning muscle attachments [24]. Some or all of these attachments may have been damaged by the fracture. The surgeon should use techniques to try and restore as much stability as possible. These include

- Head size - minimum 32 mm to increase jump distance or consider a dual mobility liner
- Accurate recreation of version, leg length and offset
- Reattachment of greater trochanter
- Muscle repair

The extra capsular fracture line is below the normal cut level of a total hip replacement. Therefore primary proximal loading cementless stems are contraindicated. The surgeon must decide if they can use a standard primary cemented stem, or must go for a distal bearing stem in order to bypass the area of bone loss. Rotational stability of a cemented stem in these osteoporotic patients usually requires a calcar for support. For this reason, distal-bearing cementless stems are commonly used. The surgeon should be experienced in these prostheses, as operations for acute failed fixation are more difficult than in the elective situation. The femur is commonly a Dorr type C (stovepipe) with an ill-defined isthmus [25]. In such osteoporotic patients there will be a higher rate of intra and post-operative fracture. Careful, accurate reaming is essential. If the greater trochanter has fractured off, then an attempt should be made at reattaching it. This can be done by the use of a trochanteric attachment plate or K-wire and cerclage technique. The muscle sling between Gluteus medius and Vastus lateralis should be carefully preserved. Attempts at reattaching the lesser trochanter are difficult and do not add much to stability or function.

Unless the metalwork has cut into the acetabulum and has produced a large defect, standard primary implants, both cemented and cementless can be used. Rarely, a large defect will have been incurred, necessitating the use of a revision shell [26]. Again care must be taken in impacting cementless shells into osteoporotic bone.

### 4.1 Late Failure

It is not uncommon for patients with painful internal fixation of extracapsular hip fractures to present to an elective hip clinic. By this stage, the fracture has usually healed, and the hip collapsed. Usually there is damage to the socket and secondary degenerative disease. By this stage, the only operative solution is a hip replacement [27]. Infection needs to be excluded. The surgeon has a choice of whether to perform this in a single stage procedure, with removal of metalwork, and then total hip replacement, or in 2 separate stages. It is our preference to perform this in one stage.

The implant is removed and this may usually be replaced with a standard primary hip implant. The anatomy is however usually abnormal; the greater trochanter may have moved anteriorly, and the lesser trochanter may be missing. Accurate templating is useful in accurately restoring patient’s leg length.

When using a cemented stem, the old screws can be inserted a few turns into the femur to stop the egress of cement during pressurisation. It is essential to try and remove extra-osseous cement after cementation.

As with a hip replacement for early failure, standard primary implants may be used for the acetabular side.

## CONCLUSION

There is neither one cause nor one solution to failed treatment of these common fractures. Surgeons need to take an accurate history, examination and then discuss options with the patient and their family. This article is not exhaustive in the methods of treatment. Ideally, revision treatment should be provided by a specialist surgeon/unit with adequate medical and rehabilitation back up. Care should be taken to get it right the second time!

## Figures and Tables

**Fig. (1):**
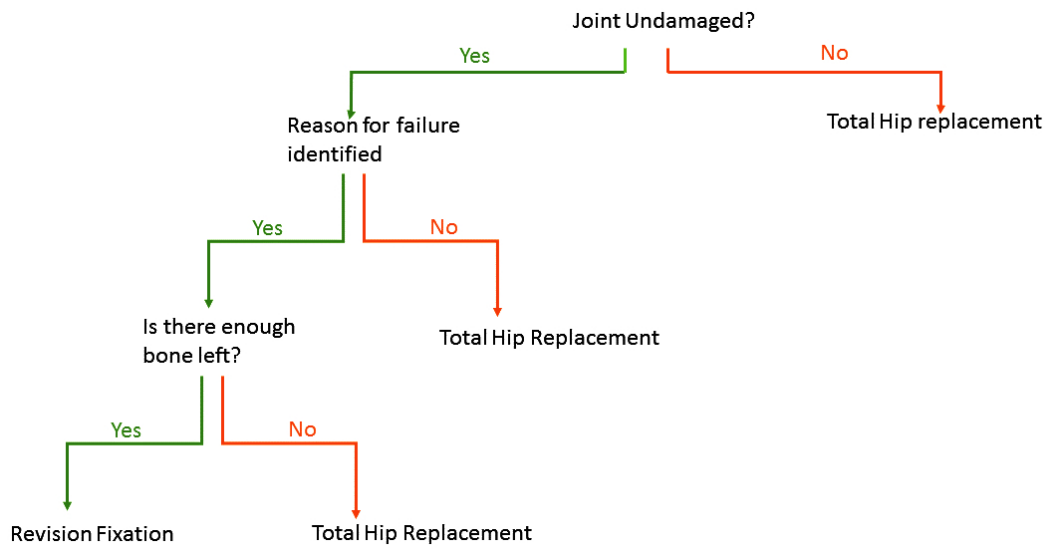
An algorithm to aid decision on whether to revise failed fixation or proceed to total hip replacement.

## LIST OF ABBREVIATIONS

DHS: = Dynamic Hip Screw
PFNA: = Proximal Femoral Nail Alpha
